# Efficacy of Enhanced Rehabilitation Initiated After Hospital Discharge to Improve Quality of Life in Survivors of Critical Care: A Systematic Review and Meta-Analysis of Randomized Controlled Trials

**DOI:** 10.7759/cureus.75184

**Published:** 2024-12-05

**Authors:** Motohiro Shimizu, Shodai Yoshihiro, Shinichi Watanabe, Gen Aikawa, Yoshihisa Fujinami, Yusuke Kawamura, Ayaka Matsuoka, Nobuto Nakanishi, Haruka Shida, Kensuke Sugimoto, Shunsuke Taito, Shigeaki Inoue

**Affiliations:** 1 Department of Intensive Care Medicine, Ryokusen-kai Yonemori Hospital, Kagoshima, JPN; 2 Department of Pharmacy, Hiroshima University Hospital, Hiroshima, JPN; 3 Department of Systematic Reviewers, Scientific Research WorkS Peer Support Group (SRWS-PSG), Osaka, JPN; 4 Department of Physical Therapy, Gifu University of Health Sciences, Gifu, JPN; 5 College of Nursing, Kanto Gakuin University, Yokohama, JPN; 6 Department of Emergency Medicine, Kakogawa Central City Hospital, Kakogawa, JPN; 7 Department of Rehabilitation, Showa General Hospital, Kodaira, JPN; 8 Department of Emergency and Critical Care Medicine, Saga University Hospital, Saga, JPN; 9 Department of Emergency and Critical Care Medicine, Tokushima University Hospital, Tokushima, JPN; 10 Department of Data Science, Medical Division, AstraZeneca K.K., Osaka, JPN; 11 Department of Intensive Care Unit, Gunma University Hospital, Maebashi, JPN; 12 Department of Clinical Practice and Support, Hiroshima University Hospital, Hiroshima, JPN; 13 Department of Emergency and Critical Care Medicine, Wakayama Medical University, Wakayama, JPN

**Keywords:** critical illness, medical critical care, post-intensive care syndrome (pics), quality of life, rehabilitation

## Abstract

This systematic review and meta-analysis evaluated the effects of enhanced rehabilitation initiated after hospital discharge on the quality of life (QOL) in survivors of critical care. The review followed the Preferred Reporting Items for Systematic Reviews and Meta-Analyses (PRISMA) procedure. MEDLINE, CENTRAL, Ichushi, Embase, PEDro, and Cumulated Index to Nursing and Allied Health Literature (CINAHL) databases and the International Clinical Trials Registry Platform and ClinicalTrials.gov (for ongoing or unpublished trials) were searched till January 2024. We identified randomized controlled trials (RCTs) with intensive care unit (ICU) survivors focusing on the effects of enhanced rehabilitation initiated after hospital discharge. Enhanced rehabilitation encompasses protocolized programs offering more intensive, frequent, or longer sessions than standard care. Primary outcomes were physical and mental components of the summary of the standardized QOL scale (SF-36) and adverse events. We calculated pooled-effect estimates for these components, expressing the mean difference (MD) and 95% confidence interval (CI). Risk of bias was evaluated using the Risk of Bias 2 tool. Certainty of evidence was assessed using the Grading of Recommendations Assessment, Development, and Evaluation (GRADE) approach. Nine RCTs (573 patients) were included. Enhanced rehabilitation resulted in no difference in the physical component-summary score (two studies: n=79, MD=3.03, 95% CI: -1.37 to 7.43, I^2^=0%, low-certainty evidence) and a higher mental component-summary score (two studies: n=79, MD=7.27, 95% CI: 2.08-12.46, I^2^=0%, low-certainty evidence). The evidence on the effect of enhanced rehabilitation on adverse events was very uncertain (nine studies: n=558, risk difference: 0.04, 95% CI: 0.00-0.07, I^2^=65%, very low-certainty evidence). Seven studies reported no adverse event occurrence, one reported a serious event requiring hospitalization in the intervention group, and another reported a minor event in the intervention group with none in controls. Enhanced rehabilitation initiated after hospital discharge may improve the mental component of QOL for survivors in the critical care. Due to the smaller number of studies included, the results need further confirmation.

## Introduction and background

Survivors of critical care frequently experience a diminished quality of life (QOL), which may persist or worsen over several years after hospital discharge [1-4]. In particular, individuals recovering from severe sepsis [3,5] and acute respiratory distress syndrome [4] or requiring complex care [3] have lower QOL scores than the general population for up to five years. Long-term impairments in physical and cognitive functions and mental health, collectively termed post-intensive care syndrome (PICS), also contribute to a reduced QOL [3]. The PICS is defined as a new or worsening impairment in physical, cognitive, and/or mental health status arising after critical illness and persisting beyond acute care discharge [6]. Moreover, survivors of critical care may face additional complications such as heterotopic ossification [7] and dysphagia [8], which further impact their QOL and recovery process. As mortality rates for critically ill patients continue to improve, the focus on QOL enhancement and PICS symptom alleviation in survivors of critical care has become increasingly important.

Rehabilitation for survivors of critical care takes place at different stages in their patient journey: during intensive care unit (ICU) stay; after ICU discharge, which represents transfer to general wards; or after hospital discharge, which represents departure from the hospital to the community. To date, systematic reviews that specifically focus on enhanced rehabilitation programs initiated after hospital discharge are scarce. Although early interventions, such as exercise training and mobilization in the intensive care unit (ICU), have shown potential in improving QOL [9] as evaluated by the Medical Outcomes Study 36-Item Short Form Health Survey (SF-36) [10] and the physical aspects of PICS [11] during hospital discharge, the long-term effectiveness of these interventions on QOL and PICS remains unclear [12-14]. A Cochrane systematic review investigated randomized controlled trials (RCTs) assessing the effects of augmented exercise rehabilitation following ICU discharge in adult survivors of critical care; however, a meta-analysis was not conducted owing to variation in study design, intervention type, and the selection and reporting of outcome measurements [15]. Although a previous systematic review [16] assessed the effects of post-ICU rehabilitation on QOL, the studies included patients who received intensive rehabilitation during hospitalization. Connolly et al. conducted a systematic review focusing on rehabilitation following hospital discharge; however, among the 10 included studies, five initiated rehabilitation during hospitalization, and two of the remaining five were study protocols without reported results [17]. Patsaki et al. also conducted a systematic review on post-hospital discharge rehabilitation, including nine studies, one of which began rehabilitation during hospitalization, and no meta-analysis was conducted [18].

While the effectiveness of rehabilitation at various stages remains inconclusive, no meta-analysis has been conducted to consolidate the findings of rehabilitation programs initiated after hospital discharge. This becomes particularly relevant because the QOL of discharged patients has been observed to improve within the first six months following hospital discharge [3].

Objective

This systematic review and meta-analysis aimed to evaluate the effectiveness of enhanced rehabilitation programs as protocolized rehabilitation programs, which are more intensive, frequent, or longer than standard care, initiated after hospital discharge on clinically important outcomes for survivors of critical care.

## Review

Protocols and registration

The review protocol was registered in the International Prospective Register of Systematic Reviews (PROSPERO) (reference number: CRD42022380429). The procedure followed the Preferred Reporting Items for Systematic Reviews and Meta-Analyses (PRISMA) statement and checklist (Appendices) [19].

Search strategy

We conducted a comprehensive literature search in December 2022 and updated it in January 2024. Initial searches were performed in MEDLINE via PubMed, Cochrane Central Register of Controlled Trials, and Igaku-Chuo-Zasshi, with all searches up to December 2, 2022. In the January 2024 update, we included additional databases, such as Embase, PEDro, and Cumulated Index to Nursing and Allied Health Literature (CINAHL), to broaden our search. We also searched for ongoing or unpublished trials in the World Health Organization International Clinical Trials Platform Search Portal and ClinicalTrials.gov databases, initially up to May 26, 2023, with an update in January 2024. The key search terms are listed in the Appendices. Our search was limited to studies published in English or Japanese language. Additionally, reference lists of international guidelines (Surviving Sepsis Campaign Guidelines 2021 [20]) and eligible studies and articles citing eligible studies were assessed. The authors of original studies were requested to provide additional or unpublished data.

Data extraction

Initially, we removed duplicates from records obtained from each database and combined them into a single dataset. Following this, two independent researchers (MS and SW) screened the records, focusing on titles and abstracts to determine preliminary eligibility. After this initial screening, articles for which a decision regarding inclusion could not be made solely based on titles and abstracts were subjected to further review. The same researchers reviewed the full texts of these articles to determine their eligibility based on predefined criteria. On confirming the eligibility of the selected articles, we utilized a design form for systematic data extraction. The form included information on the study design, population characteristics, number of participants, age, Acute Physiology and Chronic Health Evaluation II score, intervention protocol (intervention duration and frequency), controls, and outcomes. Differences in the screening results were resolved by discussion; if this failed, a third reviewer (SY) acted as an arbiter. If the relevant data were missing, the original authors were contacted.

Eligibility criteria

Study Type

RCTs were included, and non-randomized and observational studies were excluded.

Population

The population included adult survivors (≥18 years of age) admitted to medical or surgical ICU, regardless of their primary diagnosis, including COVID-19.

Intervention

The protocolized rehabilitation program included any one or more of the following: physical, occupational, speech/swallowing, respiratory, or cognitive rehabilitation. This program was initiated after hospital discharge and was designed to be more intensive, frequent, or longer than the care provided to the control group. Studies were excluded if patients in the intervention group received more intensive, frequent, or longer durations of rehabilitation during their hospital stay defined from ICU admission to acute hospitalization discharge than those in the control group, or if the intervention was provided in any inpatient setting (including but not limited to rehabilitation hospitals, neurological wards, or other medical facilities requiring overnight stay). Studies characterized as general post-ICU follow-up programs, especially those without specific rehabilitation, were also excluded.

Control

The control group received standard care or no rehabilitation after hospital discharge.

Outcomes

The primary outcomes were as follows: (1) physical component of the standardized QOL scale using the physical component summary (PCS) score of the SF-36 [10], 12-Item Short Form Health Survey (SF-12) [21], or 8-Item Short Form Health Survey (SF-8) [22]; (2) mental component of the standardized QOL scale using the mental component summary (MCS) score of the SF-36 [10], SF-12 [21], or SF-8 [22]; and (3) all adverse events defined by the original authors. We selected these QOL measures as primary outcomes because they are widely used and established tools for QOL assessment and are frequently adopted as outcome measures in clinical trials involving survivors of critical care, making them feasible for systematic review and meta-analysis. The secondary outcomes included the following: (1) physical function-related outcomes (cardiopulmonary exercise test parameters: peak oxygen consumption, peak V̇O_2_, and anaerobic threshold [23] and standardized physical function-related scale combined with six-minute walk test [24], incremental shuttle walk test [25], timed up-and-go test [26], Berg balance test [27], Short Musculoskeletal Function Assessment [28], and Rivermead Mobility Index [29]); (2) depression-related outcomes using the Hospital Anxiety and Depression Scale-Depression (HADS-D) [30], Patient Health Questionnaire-9 [31], Major Depression Inventory [32,33], and Beck Depression Inventory [34]; (3) cognitive function-related outcomes: Mini-Mental Status Examination (MMSE) [35], Modified Telephone Interview for Cognitive Status [36], and Tower test [37]; and (4) all-cause mortality. We evaluated the overall outcomes during the first six months after hospital discharge. For all outcomes, in case of multiple measurements during the first six months after hospital discharge, we extracted the one closest to six months.

Risk of bias assessment

We used version 2 of the Cochrane Risk of Bias tool for randomized trials (RoB 2) to assess the quality of the study design and the degree of potential bias according to the domains of this bias tool [38]. Two reviewers (MS and SW) independently assessed the risk of bias. Any conflicts between the two reviewers were discussed, and a third reviewer (SY) acted as an arbiter.

Data synthesis

A meta-analysis was performed using the random-effects model with the Review Manager software (RevMan version 5.4.1, The Cochrane Collaboration 2020, The Nordic Cochrane Centre, Copenhagen, Denmark). The mean differences (MDs) and the 95% confidence interval (CI) were used for the following continuous variables: PCS score, MCS score, and depression- and cognition-related outcomes. We converted the median (interquartile range) to the mean (standard deviation) using a validated method for consistent data analysis [39]. For physical function-related outcomes, as pre-specified in our protocol registered in PROSPERO, we pooled the effect estimates using standardized mean differences (SMDs) because several different scales were used across the eligible studies to measure physical function. The SMD expresses the size of the intervention effect in each study relative to the variability observed in that study, allowing us to standardize the results of studies into a uniform scale before combining them. This methodology aligns with other major systematic reviews in critical care, including the Surviving Sepsis Campaign Guidelines 2021 [20]. Relative risk ratios and 95% CI were estimated for the following binary variables: adverse events and mortality. Concerning adverse events, the risk difference with a 95% CI was evaluated as MA-MZ analysis to address the issue of a large number of included studies with zero events, which made many risk ratios inestimable [40].

Assessment of heterogeneity

We evaluated the statistical heterogeneity among eligible studies by visual inspection of the forest plots and by estimating the I2 statistic (I^2^ values of 0%-40%, could not be important; 30%-60%, moderate heterogeneity; 50%-90%, substantial heterogeneity; and 75%-100%, considerable heterogeneity) [41,42]. In case of substantial heterogeneity (I^2^>50%), the reason for heterogeneity was investigated. The Cochrane chi-squared test (Q-test) for the I^2^ statistic was performed, and a P value of <0.10 was considered statistically significant.

Rating the quality of evidence using the Grading of Recommendations Assessment, Development, and Evaluation (GRADE) approach

Two reviewers (MS and SW) independently evaluated the certainty of evidence based on the GRADE approach [43]. The quality of the body of evidence was assessed for each outcome and categorized as high, moderate, low, or very low using the GRADE Pro Guideline Development Tool. Disagreements between the two reviewers were discussed, and if they remained unresolved, a third reviewer (SY) acted as the arbiter.

Subgroup analyses

We performed subgroup analyses based on the primary outcomes of the studies according to the duration of the intervention (≤8 and >8 weeks) and treatment frequency (<3 and ≥3 times a week). We also planned to perform a subgroup analysis targeting older adults (those ≥65 years of age) but could not proceed due to insufficient data.

Sensitivity analyses

Sensitivity analyses of the primary outcomes were performed to examine whether the results of the review and meta-analysis were robust to decisions made during the review process. The results were compared to exclude studies that used imputed statistics.

Results

Search Results

**Figure 1:**
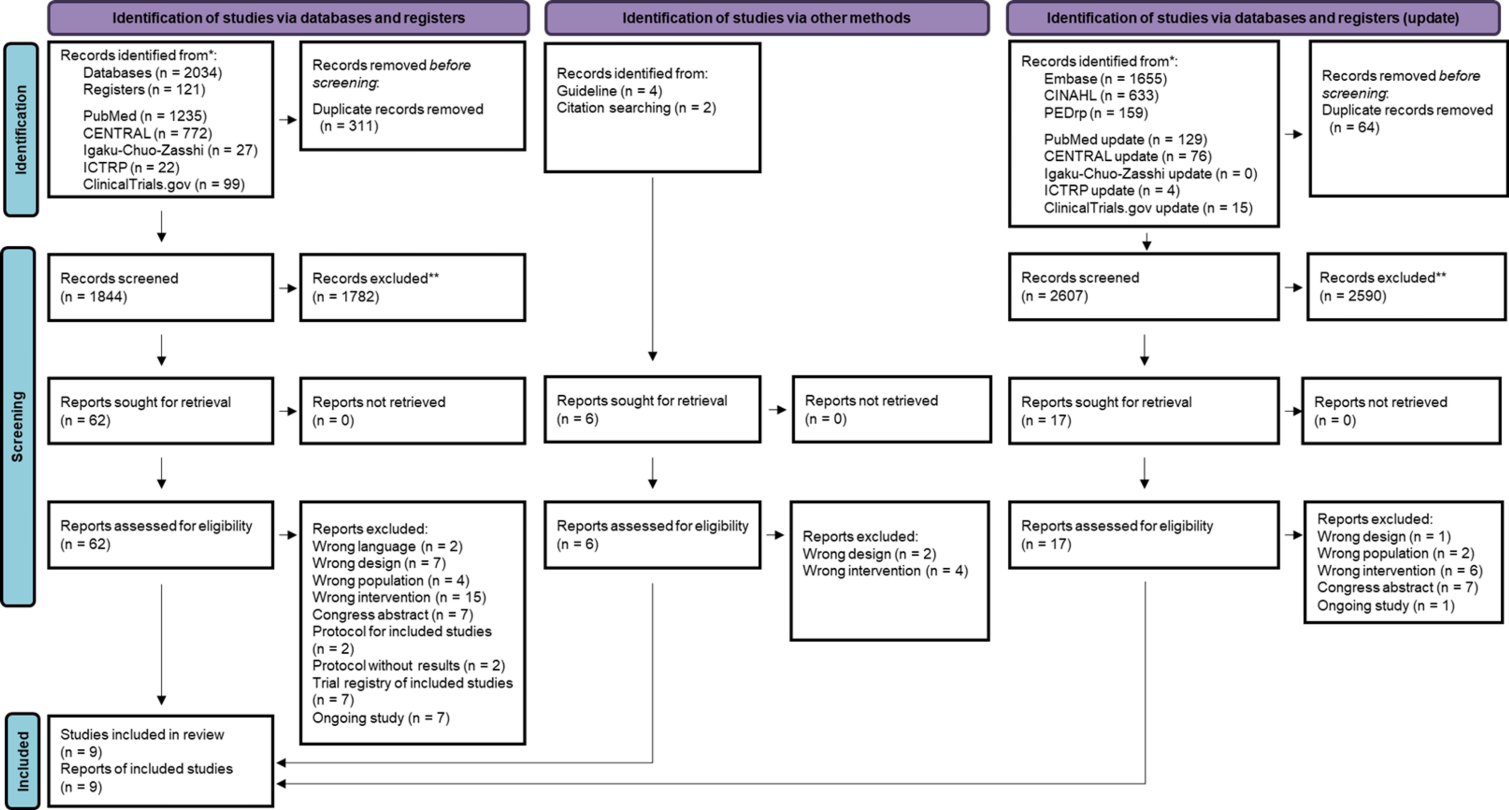
PRISMA 2020 flow diagram PRISMA: Preferred Reporting Items for Systematic Reviews and Meta-Analyses, ICTRP: International Clinical Trials Registry Platform, CINAHL: Cumulated Index to Nursing and Allied Health Literature

The PRISMA flowchart depicting the selection of studies to be included in this meta-analysis is displayed in Figure 1.

Our search strategy initially yielded 1844 citations. Of these, 62 citations were considered potentially eligible based on their abstracts and were subjected to a full-text review. Following the full-text review, 53 citations were excluded (Appendices), and nine RCTs [44-52] met the eligibility criteria for inclusion in our review. In the January 2024 update of our search, which was expanded to include additional databases not initially reviewed and capture new publications, we identified an additional 2594 records. Seventeen of these were selected for full-text screening, but none met our inclusion criteria and were excluded (Appendices). Furthermore, we assessed the reference lists of international guidelines and eligible studies and articles citing eligible studies. Four studies were identified for full-text evaluation based on the screening of international guidelines. Two additional studies were identified by screening the references and cited articles of eligible studies. After evaluating the full texts, all six studies were excluded.

Characteristics of Included Studies

Nine studies from five countries (UK, Australia, India, Italy, and USA) with a total of 573 participants (288 in the intervention group and 285 in the control group) were included in this review. Descriptions of the cohorts and specific study interventions are listed in Table 1.

**Table 1:** Characteristics of included studies RCT: randomized controlled trial, I: intervention group, C: control group, ICU: intensive care unit, LOS: length of stay, QOL: quality of life, SF-36: 36-Item Short Form Health Survey, 6MWT: 6-minute walk test, TUG: timed up-and-go test, MMSE: Mini-Mental Status Examination, EQ-5D: EuroQol five-dimension questionnaire, HADS: Hospital Anxiety and Depression Scale, ISWT: incremental shuttle walk test, ADL: activities of daily living, MRC: Medical Research Council

Author, year, country	Study type	Number of participants		Age (mean or median)		ICU LOS days (median)		Intervention (a: contents, b: duration, c: frequency)	Control	Outcomes (a: assessment tools, b: assessment timepoint)
		I	C	I	C	I	C			
Elliott et al., 2011, Australia [44]	Multicenter RCT	97	98	57.2	57.5	6	6	a: Home-based program with structured strength training and walking exercises, focusing on improving physical mobility and endurance, b: 8 weeks, c: 5 times/week	Usual care (no intervention)	a: QOL (SF-36), physical function-related outcomes (6MWT), adverse events, mortality, b: 26 weeks after hospital discharge
Jackson et al., 2012, USA [45]	Single-center pilot RCT	13	8	47	50	3	5.8	a: Comprehensive in-home program encompassing cognitive rehabilitation and physical therapy exercises tailored to individual patient needs, b: 12 weeks, c: 1 time/week	Usual care (no intervention)	a: Physical function-related outcomes (TUG), cognitive function-related outcomes (MMSE, tower test), adverse events, mortality, b: 3 months after hospital discharge
Batterham et al., 2014, UK [46]	Multicenter RCT	29	30	42.7	40.5	15	15	a: Physiotherapist-led, hospital-based exercise sessions focused on improving cardiorespiratory fitness and muscle strength, b: 8 weeks, c: 2 times/week	Usual care (no intervention)	a: QOL (SF-36, EQ-5D), cardiopulmonary exercise test, depression symptoms (HADS), adverse events, mortality, b: 26 weeks after hospital discharge
Connolly et al., 2015, UK [47]	Two-center pilot RCT	10	10	63	68.5	14.5	13.0	a: A combination of supervised and unsupervised exercise sessions aimed at enhancing overall physical fitness and mobility, b: 8 weeks, c: 3 times/week (2 times supervised, 1 time unsupervised)	Usual care (no intervention)	a: QOL (SF-36), physical function-related outcomes (6MWT, ISWT, TUG), muscle size and strength, anxiety and depression symptoms (HADS), ADL, adverse events, mortality, b: 3 months after hospital discharge
McWilliams et al., 2016, UK [48]	Single-center RCT	37	36	55	60.8	29.1	22.2	a: Outpatient program integrating exercise with educational components, designed to improve both physical capability and knowledge about self-care, b: 7 weeks, c: 3 times/week (1 supervised, 2 self-directed titrated)	Usual care (no intervention)	a: QOL (SF-36), cardiopulmonary exercise test, adverse events, mortality, b: N/A
Vitacca et al., 2016, Italy [49]	Single-center RCT	24	24	68.25	63	36	20	a: Home-based program with a focus on pulmonary rehabilitation, including daily exercises for breathing and muscle strength, under caregiver supervision, b: 6 months, c: every day	Usual care (no intervention)	a: QOL (EQ-5D), pulmonary function, arterial blood gas values, ADL, MRC scale, adverse events, mortality, b: 6 months after hospital discharge
Shelly et al., 2017, India [51]	Single-center RCT	17	18	59	53	8	9.5	a: Home-based training emphasizing respiratory function and mobility through a series of tailored exercises, b: 4 weeks, c: 5 times/week	Usual care (no intervention)	a: QOL (SF-36), adverse events, mortality, b: 4 weeks after hospital discharge
McDowell et al., 2017, UK [50]	Multicenter RCT	30	30	51	51	16.0	13.0	a: Standard care supplemented with a personalized exercise program, including both supervised and unsupervised sessions, b: 6 weeks, c: 3 times/week (2 supervised and 1 unsupervised)	Usual care (no intervention)	a: QOL (SF-36), physical function-related outcomes (ISWT, Rivermead Mobility Index), hand function, readiness to exercise, self-efficacy to exercise, anxiety and depression symptoms (HADS), breathlessness, adverse events, mortality, b: 6 weeks and 6 months post-intervention
Battle et al., 2019, UK [52]	Single-center RCT	31	31	61	62.5	12	7	a: Individually tailored exercise program, supervised by professionals, designed to enhance cardiorespiratory fitness and physical strength, b: 6 weeks, c: 2 times/week	Usual care (no intervention)	a: Physical function-related outcomes (6MWT, Berg Balance Test), depression symptoms (HADS), adverse events, mortality, b: 7 weeks, 6 and 12 months post-intervention onset

In the included studies, post-hospital rehabilitation programs for ICU survivors included a combination of activities, including cardiorespiratory exercises such as cycling and walking, diverse strength and balance training with equipment, and specific mobility exercises to enhance daily activities. Additionally, some programs integrated pulmonary rehabilitation with breathing and muscle strength exercises, along with cognitive and physical therapies. The duration of these programs ranged from four weeks to six months.

Primary Outcomes

**Figure 2:**
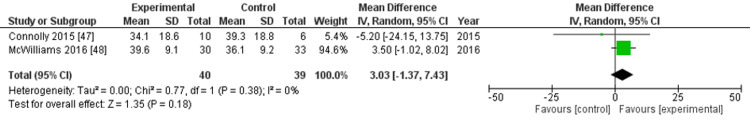
Forest plot for outcomes: physical component summary

Physical and mental component summaries: Two RCTs [47,48] reported physical and mental component summary scores of the SF-36 as outcomes. Enhanced rehabilitation initiated after hospital discharge may have minimal to no difference in the PCS score (MD=3.03, 95% CI: -1.37 to 7.43, P=0.18, I^2^=0%, two studies, 79 participants, low-certainty evidence) (Figure 2, Table 2).

**Table 2:** Summary of findings ^*^The risk in the intervention group (and its 95% confidence interval) is based on the assumed risk in the comparison group and the relative effect of the intervention (and its 95% CI). CI: confidence interval, MD: mean difference, RR: risk ratio, SMD: standardized mean difference, PCS: physical component summary, MCS: mental component summary Explanations ^a^Downgraded one level owing to a high risk of bias across varying domains ^b^Downgraded one level owing to the insufficient sample size ^c^Downgraded one level owing to high heterogeneity ^d^Downgraded three levels because of the high risk of bias across varying domains and lack of reported data from eight studies ^e^Downgraded one level owing to imprecision due to wide confidence intervals that cross the line of null effect GRADE Working Group grades of evidence High certainty: very confident that the true effect lies close to that of the estimate of the effect; moderate certainty: moderately confident in the effect estimate: the true effect is likely to be close to the estimate of the effect, with a possibility that it is substantially different; low certainty: limited confidence in the effect estimate: the true effect may be substantially different from the estimate of the effect; very low certainty: very limited confidence in the effect estimate: the true effect is likely to be substantially different from the estimate of the effect

Post-hospital rehabilitation compared to usual care in survivors of critical care						
Patient or population: survivors of critical care, setting: home, intervention: post-hospital rehabilitation, comparison: usual care						
Outcomes	Anticipated absolute effects^*^ (95% CI)		Relative effect (95% CI)	Number of participants (studies)	Certainty of the evidence (GRADE)	Comments
	Risk usual care	Risk rehabilitation				
PCS	-	MD 3.03 higher (1.37 lower to 7.43 higher)	-	79 (2 RCTs)	⨁⨁◯◯ Low ^a,b^	-
MCS	-	MD 7.27 higher (2.08 higher to 12.46 higher)	-	79 (2 RCTs)	⨁⨁◯◯ Low ^a,b^	-
All adverse events	11 per 1,000	51 per 1,000 (11 to 81)	-	558 (9 RCTs)	⨁◯◯◯ Very low ^a,b,c^	-
Physical function-related outcomes	-	SMD 0.14 higher (0.21 lower to 0.48 higher)	-	131 (4 RCTs)	⨁⨁◯◯ Low ^a,b^	-
Cognitive function-related outcomes	Only 1 study reported cognitive function-related outcome. The intervention group had a higher median MMSE score (30.0 (interquartile range: 29.0-30.0)) compared to the control group (26.5 (interquartile range: 24.8-28.5))			15 (1 RCT)	⨁◯◯◯ Very low ^d^	-
Depression-related outcomes	-	MD 1.26 lower (3.44 lower to 0.92 higher)	-	57 (2 RCTs)	⨁⨁◯◯ Low ^a,b^	-
All-cause mortality	33 per 1,000	46 per 1,000 (20 to 107)	RR 1.42 (0.62 to 3.27)	558 (9 RCTs)	⨁⨁◯◯ Low ^a,e^	-

**Figure 3:**
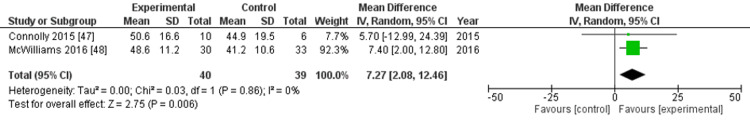
Forest plot for outcomes: mental component summary

However, the evidence suggested that enhanced rehabilitation initiated after hospital discharge may increase the MCS scores (MD=7.27, 95% CI: 2.08-12.46, P=0.006, I^2^=0%, two studies, 79 participants, low-certainty evidence) (Figure 3, Table 2).

The overall risk of bias was categorized as serious, attributable to the absence of an appropriate analysis for estimating the effect of intervention assignment, missing outcome data, and potential risk in selecting the reported results (Appendices). The imprecision was assessed as serious because of the limited sample size. Consequently, the certainty of the evidence for these outcomes was rated as low.

**Figure 4:**
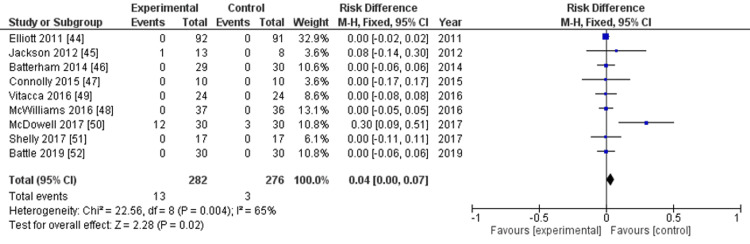
Forest plot for outcomes: all adverse events

Adverse events: Adverse events were assessed in nine RCTs [44-52]. The evidence suggested that enhanced rehabilitation initiated after hospital discharge resulted in minimal to no difference in adverse events, but the evidence is very uncertain (risk difference: 0.04, 95% CI: 0.00-0.07, P=0.02, I^2^=65%, nine studies, 558 participants, very low-certainty evidence) (Figure 4, Table 2).

To address the issue of a large number of included studies with zero events, making many risk ratios not estimable, we employed the Mantel-Haenszel method to evaluate the risk difference as MA-MZ analysis [40]. The sensitivity analysis of the exact p-function methods confirmed the result (risk difference: 0.04, 95% CI: 0.01-0.06). The overall risk of bias was classified as serious (Appendices). The rating for imprecision was deemed serious because the number of events did not meet the optimal information size. The inconsistency was also considered serious, primarily due to high heterogeneity (I^2^=65%). These serious risks led to the certainty of the evidence being downgraded to very low. Seven of the nine RCTs reported adverse events in neither the intervention nor the control group. In two studies [45,50], one reported 12 events in the intervention group and three in the control group; the other study recorded only one minor event in the intervention group and none in the control group. Most adverse events were minor; however, one study [50] reported a serious adverse event requiring hospitalization linked to the intervention.

Secondary Outcomes

**Figure 5:**
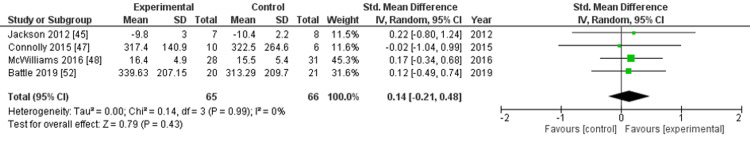
Forest plot for outcomes: physical function-related outcomes

Physical function-related outcomes: Four RCTs evaluated the effects of enhanced rehabilitation initiated after hospital discharge on physical outcomes. Two of these studies used the six-minute walk test [47,52], one measured peak V̇O_2_ [48], and another utilized the timed up-and-go test [45]. Enhanced rehabilitation initiated after hospital discharge might slightly improve physical function-related outcomes (SMD: 0.14, 95% CI: -0.21 to 0.48, P=0.43, I^2^=0%, four studies, 131 participants, low-certainty evidence) (Figure 5, Table 2).

The overall risk of bias was categorized as serious (Appendices). Imprecision was considered serious owing to the limited sample size, leading to a low level of certainty in the evidence.

Cognitive function-related outcomes: One RCT [45] assessed the effects of enhanced rehabilitation initiated after hospital discharge on MMSE, a cognitive function-related outcome. The study reported that at the end of the intervention period, the intervention group had a higher median MMSE score (30.0, interquartile range: 29.0-30.0) compared to the control group (26.5, interquartile range: 24.8-28.5). The risk of bias was labeled serious because of the absence of a suitable analysis for gauging the intervention's effect, missing outcome data, and potential issues in selecting the reported results (Appendices). Imprecision was deemed serious because of the limited number of participants in the study (15 participants). Consequently, the certainty of the evidence for this outcome was rated as very low.

**Figure 6:**
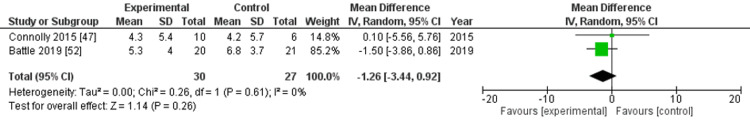
Forest plot for outcomes: depression-related outcomes

Depression-related outcomes: Two RCTs [47,52] focused on depression-related outcomes using the HADS-D. The evidence suggested that enhanced rehabilitation initiated after hospital discharge resulted in minimal to no difference in depression-related outcomes (MD: -1.26, 95% CI: -3.44 to 0.92, P=0.26, I^2^=0%, two studies, 57 participants, low-certainty evidence) (Figure 6, Table 2).

The risk of bias was labeled serious owing to the absence of a suitable analysis for gauging the effects of the intervention and missing outcome data (Appendices). The limited sample size also contributed to the imprecision being considered significant. Collectively, these factors resulted in the certainty of the evidence being downgraded to low levels.

**Figure 7:**
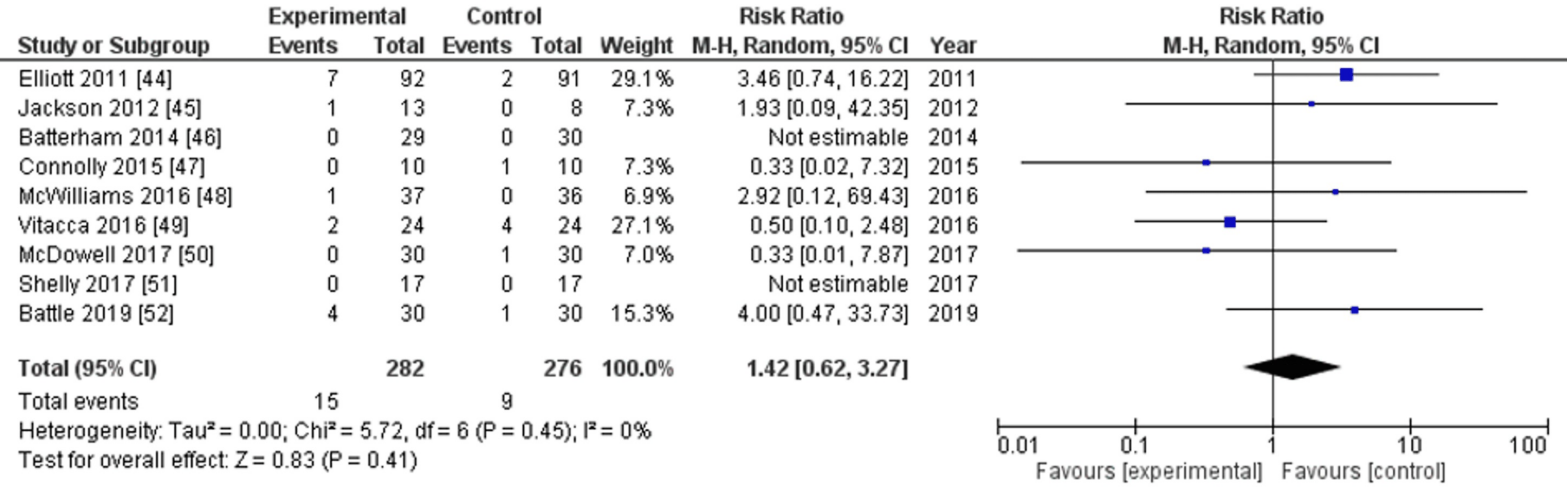
Forest plot for outcomes: all-cause mortality

All-cause mortality: Nine RCTs reported all-cause mortality [44-52]. The evidence suggested that enhanced rehabilitation initiated after hospital discharge resulted in minimal to no difference in all-cause mortality (risk ratio: 1.42, 95% CI: 0.62-3.27, P=0.41, I^2^=0%, nine studies, 558 participants, very low-certainty evidence) (Figure 7, Table 2).

The overall risk of bias was classified as serious (Appendices). The rating for imprecision was deemed serious because of the wide CI that crossed the line of no effect, resulting in the certainty of the evidence being rated low.

Subgroup Analyses

**Figure 8:**
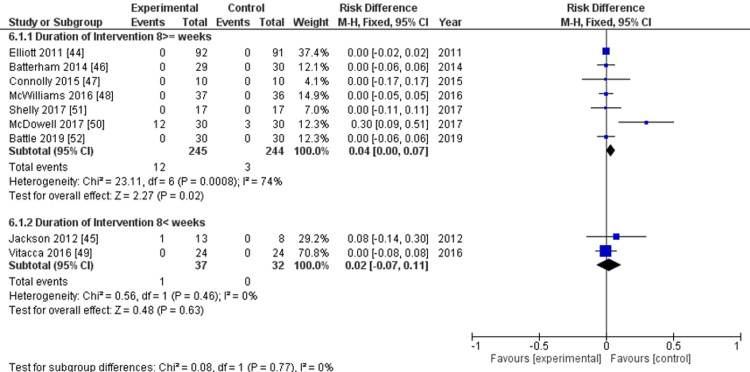
Subgroup analysis for the primary outcomes All adverse events: duration of intervention (≤8 and >8 weeks)

Subgroup analyses for PCS and MCS were not performed because both studies that evaluated these outcomes were categorized within the same subgroup. The adverse events between the two rehabilitation duration groups showed no significant differences (Figure 8). The subgroup analysis of the frequency of rehabilitation was also not conducted because the two studies that contributed data to this analysis were in the same subgroup.

Sensitivity Analysis

**Figure 9:**
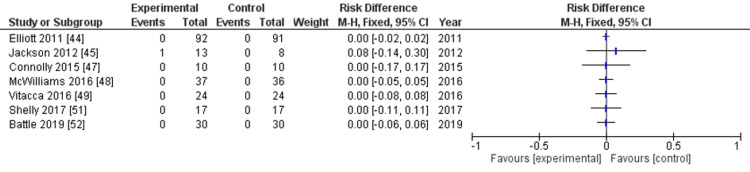
Sensitivity analyses for the primary outcomes (exclusion of studies using imputed statistics) All adverse events

We could not perform a pre-specified sensitivity analysis based on the presence of imputation because the studies assessing PCS and MCS as outcomes did not employ imputation statistics. The results of a separate sensitivity analysis that excluded studies using imputed statistics for all adverse events are shown in Figure 9.

Discussion

Our systematic review and meta-analysis suggested a slight improvement in the mental component of QOL, assessed using the SF-36, specifically by focusing on enhanced rehabilitation initiated after hospital discharge. Evidence regarding the effects of ICU rehabilitation on long-term QOL, particularly the mental component, is limited [53]. A previous systematic review and meta-analysis found that rehabilitation after ICU discharge resulted in minimal to no difference in the PCS and MCS of QOL, as evaluated either with the SF-36 or SF-12 [16]. Because the MCS includes aspects such as vitality, social functioning, role-emotional, and mental health [54], enhanced rehabilitation may be more effective in improving these elements in the home setting after discharge than during hospitalization. Therefore, emphasis on enhanced rehabilitation to improve MCS may be most appropriate at the post-hospital discharge stage. Considering the limited number of studies included in our meta-analysis, further research is required to ascertain the impact of enhanced rehabilitation after hospital discharge on the mental component of QOL.

This systematic review and meta-analysis could not determine whether enhanced rehabilitation initiated after hospital discharge increases adverse events or all-cause mortality. Adverse events were thoroughly assessed across the nine studies evaluated in our systematic review. Seven studies reported no adverse events in the intervention and control groups, while two studies reported such events. The reported events were predominantly minor, although one study noted a serious event requiring hospitalization linked to the intervention. These results emphasize the need for careful monitoring and tailored approaches in post-discharge rehabilitation and suggest that enhanced rehabilitation does not increase the risk of severe, life-threatening complications for survivors of critical care. In contrast, enhanced rehabilitation during ICU stays increases the incidence of adverse events [55]. Enhanced rehabilitation can influence the occurrence of adverse events, even after hospital discharge. Further studies are required to explore the potential benefits and risks of enhanced rehabilitation programs.

Methods for enhanced rehabilitation to improve QOL, physical and cognitive functions, and mental health are diverse, and the optimal approach remains unclear. Interventions for enhanced rehabilitation initiated after hospital discharge vary widely across the studies in our systematic review and meta-analysis, reflecting diverse methodologies. Some studies have focused on supervised physiotherapy sessions, others on individualized exercise programs, and others on home-based or technology-assisted interventions. The control groups in these studies generally received standard care with some variations. This diversity underscores the multifaceted nature of rehabilitation strategies and the ongoing exploration of optimal approaches to improve QOL in survivors of critical care. Differences in the frequency, duration, and style of interventions highlight the need for further studies to determine the most effective rehabilitation methods after hospital discharge.

The included studies in our systematic review and meta-analysis demonstrated significant heterogeneity in the components and delivery methods of enhanced rehabilitation programs initiated after hospital discharge. These programs ranged from structured physiotherapy sessions [44,46] to self-managed exercise programs [48] and from supervised face-to-face interventions [52] to home-based rehabilitation [49]. The frequency of sessions varied from daily [49] to weekly [45], and the duration of programs ranged from four weeks [51] to six months [49]. This diversity in intervention components and delivery methods reflects the current lack of standardization in enhanced rehabilitation initiated after hospital discharge for survivors of critical care. While this variety might allow for individualized approaches, it makes it challenging to determine the most effective components and the optimal "dose" of rehabilitation. A formal and structured care pathway may more appropriately address patients' comprehensive rehabilitation needs [56]. Future research should focus on identifying which specific components, frequency, and intensity of enhanced rehabilitation are most beneficial for different patient populations.

This systematic review has some limitations that must be considered when interpreting the findings. First, the number of included studies and the sample sizes of some studies were small, which limited the scope of our findings and our ability to conduct the planned subgroup and sensitivity analyses. Second, the interventions in the included studies were diverse, covering both physical and cognitive rehabilitation. While representative of clinical practice, this variety makes it challenging to draw definitive conclusions on the broad outcomes assessed. As more studies become available, conducting analyses that evaluate the effects of specific types of interventions individually will be possible. Third, most studies evaluated the outcomes immediately after the end of the intervention. Therefore, whether the improvement in outcomes from enhanced rehabilitation after hospital discharge is maintained in the long term is to be determined. Fourth, the included studies did not consistently report or analyze whether patients were discharged directly from the ICU or after transfer to a general ward. This discharge pathway could potentially influence rehabilitation outcomes, but the current evidence base does not allow for separate analyses based on these discharge patterns. Fifth, the included studies were conducted across some different countries with varying healthcare systems, and variations in the length of hospital stay and rehabilitation protocols among studies were not consistently reported. These differences could influence outcomes, but the limited number of studies precluded analyses of their impact. The studies also showed variety in supervision levels and settings, from fully supervised to partially supervised programs, and facility-based to home-based interventions. While we had not planned subgroup or sensitivity analyses for these factors, they should be considered in future studies. Sixth, our review was restricted to studies published in English or Japanese, which may introduce language bias. Seventh, although we planned to assess outcomes using various tools, only a limited number of tools were reported in the included studies, which restricted our ability to comprehensively evaluate the effects of enhanced rehabilitation on different aspects of the outcomes.

## Conclusions

In conclusion, enhanced rehabilitation initiated after hospital discharge may result in minimal to no difference in the physical component but may slightly improve the mental component of QOL for survivors of critical care. The analysis also indicates minimal to no increase in adverse events or all-cause mortality, underscoring the necessity for careful monitoring and individualized rehabilitation approaches. Further studies are required to understand the factors that contribute to the improvement of the mental component of QOL.

## Figures and Tables

**Figure 10:**
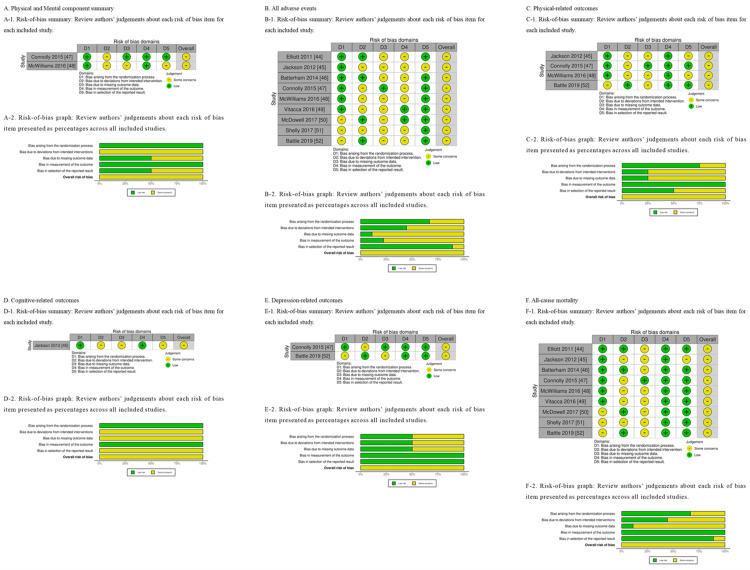
Risk of bias summary and graph A: physical and mental component summary, B: all adverse events, C: physical-related outcomes, D: cognitive-related outcomes, E: depression-related outcomes, F: all-cause mortality

## Appendices

Table 3 and Table 4 show the PRISMA checklist followed in this study.

**Table 3:** PRISMA 2020 checklist PRISMA: Preferred Reporting Items for Systematic Reviews and Meta-Analyses From: Page MJ, McKenzie JE, Bossuyt PM, Boutron I, Hoffmann TC, Mulrow CD, et al. The PRISMA 2020 statement: an updated guideline for reporting systematic reviews. BMJ. 2021;372:n71. https://doi.org/10.1136/bmj.n71

Section and topic	Item #	Checklist item	Location where item is reported
Title			
Title	1	Identify the report as a systematic review.	Page 1
Abstract			
Abstract	2	See the PRISMA 2020 for abstracts checklist.	Appendix Table 4
Introduction			
Rationale	3	Describe the rationale for the review in the context of existing knowledge.	Page 2-3
Objectives	4	Provide an explicit statement of the objective(s) or question(s) the review addresses.	Page 3
Methods			
Eligibility criteria	5	Specify the inclusion and exclusion criteria for the review and how studies were grouped for the syntheses.	Page 4-5
Information sources	6	Specify all databases, registers, websites, organizations, reference lists and other sources searched or consulted to identify studies. Specify the date when each source was last searched or consulted.	Page 3-4
Search strategy	7	Present the full search strategies for all databases, registers and websites, including any filters and limits used.	Appendix Table 5-12
Selection process	8	Specify the methods used to decide whether a study met the inclusion criteria of the review, including how many reviewers screened each record and each report retrieved, whether they worked independently, and if applicable, details of automation tools used in the process.	Page 4
Data collection process	9	Specify the methods used to collect data from reports, including how many reviewers collected data from each report, whether they worked independently, any processes for obtaining or confirming data from study investigators, and if applicable, details of automation tools used in the process.	Page 4-6
Data items	10a	List and define all outcomes for which data were sought. Specify whether all results that were compatible with each outcome domain in each study were sought (e.g. for all measures, time points, analyses), and if not, the methods used to decide which results to collect.	Page 5
	10b	List and define all other variables for which data were sought (e.g. participant and intervention characteristics, funding sources). Describe any assumptions made about any missing or unclear information.	Page 4-6
Study risk of bias assessment	11	Specify the methods used to assess risk of bias in the included studies, including details of the tool(s) used, how many reviewers assessed each study and whether they worked independently, and if applicable, details of automation tools used in the process.	Page 5
Effect measures	12	Specify for each outcome the effect measure(s) (e.g. risk ratio, mean difference) used in the synthesis or presentation of results.	Page 5-6
Synthesis methods	13a	Describe the processes used to decide which studies were eligible for each synthesis (e.g. tabulating the study intervention characteristics and comparing against the planned groups for each synthesis (item #5)).	Page 6
	13b	Describe any methods required to prepare the data for presentation or synthesis, such as handling of missing summary statistics, or data conversions.	Page 6
	13c	Describe any methods used to tabulate or visually display results of individual studies and syntheses.	Page 6
	13d	Describe any methods used to synthesize results and provide a rationale for the choice(s). If meta-analysis was performed, describe the model(s), method(s) to identify the presence and extent of statistical heterogeneity, and software package(s) used.	Page 5
	13e	Describe any methods used to explore possible causes of heterogeneity among study results (e.g. subgroup analysis, meta-regression).	Page 6-7
	13f	Describe any sensitivity analyses conducted to assess robustness of the synthesized results.	Page 7
Reporting bias assessment	14	Describe any methods used to assess risk of bias due to missing results in a synthesis (arising from reporting biases).	Page 6
Certainty assessment	15	Describe any methods used to assess certainty (or confidence) in the body of evidence for an outcome.	Page 6
Results			
Study selection	16a	Describe the results of the search and selection process, from the number of records identified in the search to the number of studies included in the review, ideally using a flow diagram.	Figure 1
	16b	Cite studies that might appear to meet the inclusion criteria, but which were excluded, and explain why they were excluded.	Appendix Table 13
Study characteristics	17	Cite each included study and present its characteristics.	Table 1
Risk of bias in studies	18	Present assessments of risk of bias for each included study.	Table 2
Results of individual studies	19	For all outcomes, present, for each study: (a) summary statistics for each group (where appropriate) and (b) an effect estimate and its precision (e.g. confidence/credible interval), ideally using structured tables or plots.	Figure 2-7
Results of syntheses	20a	For each synthesis, briefly summarise the characteristics and risk of bias among contributing studies.	Page 8-10
	20b	Present results of all statistical syntheses conducted. If meta-analysis was done, present for each the summary estimate and its precision (e.g. confidence/credible interval) and measures of statistical heterogeneity. If comparing groups, describe the direction of the effect.	Page 8-10, Figure 2-7
	20c	Present results of all investigations of possible causes of heterogeneity among study results.	Page 10, Figure 10
	20d	Present results of all sensitivity analyses conducted to assess the robustness of the synthesized results.	Page 10, Figure 10
Reporting biases	21	Present assessments of risk of bias due to missing results (arising from reporting biases) for each synthesis assessed.	Page 8-10
Certainty of evidence	22	Present assessments of certainty (or confidence) in the body of evidence for each outcome assessed.	Page 8-10, Figure 2-7, Table 2
Discussion			
Discussion	23a	Provide a general interpretation of the results in the context of other evidence.	Page 11
	23b	Discuss any limitations of the evidence included in the review.	Page 12
	23c	Discuss any limitations of the review processes used.	Page 12
	23d	Discuss implications of the results for practice, policy, and future research.	Page 12
Other information			
Registration and protocol	24a	Provide registration information for the review, including register name and registration number, or state that the review was not registered.	Page 2, 3
	24b	Indicate where the review protocol can be accessed, or state that a protocol was not prepared.	Page 2, 3
	24c	Describe and explain any amendments to information provided at registration or in the protocol.	Not applicable
Support	25	Describe sources of financial or non-financial support for the review, and the role of the funders or sponsors in the review.	Page 13-14
Competing interests	26	Declare any competing interests of review authors.	Page 14
Availability of data, code, and other materials	27	Report which of the following are publicly available and where they can be found: template data collection forms; data extracted from included studies; data used for all analyses; analytic code; any other materials used in the review.	Page 14

**Table 4:** PRISMA 2020 for abstracts checklist PRISMA: Preferred Reporting Items for Systematic Reviews and Meta-Analyses

Section and topic	Item #	Checklist item	Reported (yes/no)
Title			
Title	1	Identify the report as a systematic review.	Yes
Background			
Objectives	2	Provide an explicit statement of the main objective(s) or question(s) the review addresses.	Yes
Methods			
Eligibility criteria	3	Specify the inclusion and exclusion criteria for the review.	Yes
Information sources	4	Specify the information sources (e.g., databases, registers) used to identify studies and the date when each was last searched.	Yes
Risk of bias	5	Specify the methods used to assess risk of bias in the included studies.	Yes
Synthesis of results	6	Specify the methods used to present and synthesize results.	Yes
Results			
Included studies	7	Give the total number of included studies and participants and summarize relevant characteristics of studies.	Yes
Synthesis of results	8	Present results for main outcomes, preferably indicating the number of included studies and participants for each. If meta-analysis was done, report the summary estimate and confidence/credible interval. If comparing groups, indicate the direction of the effect (i.e., which group is favored).	Yes
Discussion			
Limitations of evidence	9	Provide a brief summary of the limitations of the evidence included in the review (e.g., study risk of bias, inconsistency and imprecision).	Yes
Interpretation	10	Provide a general interpretation of the results and important implications.	Yes
Other			
Funding	11	Specify the primary source of funding for the review.	No
Registration	12	Provide the register name and registration number.	Yes

The key search terms used in the present study are listed in Table 5-12.

**Table 5:** Search strategies: MEDLINE via PubMed search strategy

Searches	Keywords
#1	"Critical Illness"[mh] OR "Critical Care"[mh] OR "critically ill"[tiab] OR "critical care"[tiab] OR "critical illness"[tiab]
#2	"physical therapy modalities"[mh] OR "occupational therapy"[mh] OR "Physical and Rehabilitation Medicine"[mh] OR "Exercise Therapy"[mh] OR Rehabilitation[mh] OR "Patient Care Planning"[mh] OR physiotherap*[tiab] OR "occupational therapy"[tiab] OR "physical therapy"[tiab] OR "Exercise Therapy"[tiab] OR Rehabilitation[tiab] OR "Patient Care Planning"[tiab]
#3	(randomized controlled trial[pt] OR controlled clinical trial[pt] OR randomized[tiab] OR placebo[tiab] OR drug therapy[sh] OR randomly[tiab] OR trial[tiab] OR groups[tiab] NOT (animals [mh] NOT humans [mh]))
#4	#1 AND #2 AND #3

**Table 6:** Search strategies: CENTRAL search strategy

Searches	Keywords
#1	MeSH descriptor: [Critical Illness] explode all trees
#2	MeSH descriptor: [Critical Care] explode all trees
#3	("critically ill"):ti,ab,kw
#4	("critical care"):ti,ab,kw
#5	("critical illness"):ti,ab,kw
#6	#1 OR #2 OR #3 OR #4 OR #5
#7	MeSH descriptor: [Physical Therapy Modalities] explode all trees
#8	MeSH descriptor: [Occupational Therapy] explode all trees
#9	MeSH descriptor: [Physical and Rehabilitation Medicine] explode all trees
#10	MeSH descriptor: [Exercise Therapy] explode all trees
#11	MeSH descriptor: [Rehabilitation] explode all trees
#12	MeSH descriptor: [Patient Care Planning] explode all trees
#13	(physiotherap*):ti,ab,kw
#14	("occupational therapy"):ti,ab,kw
#15	("physical therapy"):ti,ab,kw
#16	("Exercise Therapy"):ti,ab,kw
#17	(Rehabilitation):ti,ab,kw
#18	("Patient Care Planning"):ti,ab,kw
#19	#7 OR #8 OR #9 OR #10 OR #11 OR #12 OR #13 OR #14 OR #15 OR #16 OR #17 OR #18
#20	#6 AND #19

**Table 7:** Search strategies: Embase search strategy

Searches	Keywords
S1	EMB.EXACT.EXPLODE("Critical Illness")
S2	ab("critically ill") OR ti("critically ill")
S3	ab("critical care") OR ti("critical care")
S4	ab("critical illness") OR ti("critical illness")
S5	S1 OR S2 OR S3 OR S4
S6	EMB.EXACT.EXPLODE("physiotherapy")
S7	EMB.EXACT.EXPLODE("occupational therapy")
S8	EMB.EXACT.EXPLODE("rehabilitation medicine")
S9	EMB.EXACT.EXPLODE("kinesiotherapy")
S10	EMB.EXACT.EXPLODE("rehabilitation")
S11	EMB.EXACT.EXPLODE("patient care planning")
S12	ab(physiotherap*) OR ti(physiotherap*)
S13	ab("occupational therapy") OR ti("occupational therapy")
S14	ab("physical therapy") OR ti("physical therapy")
S15	ab("Exercise Therapy") OR ti("Exercise Therapy")
S16	ab(Rehabilitation) OR ti(Rehabilitation)
S17	ab("Patient Care Planning") OR ti("Patient Care Planning")
S18	S6 OR S7 OR S8 OR S9 OR S10 OR S11 OR S12 OR S13 OR S14 OR S15 OR S16 OR S17
S19	EMB.EXACT.EXPLODE("randomized controlled trial")
S20	EMB.EXACT.EXPLODE("controlled clinical trial")
S21	TI(random*) OR AB(random*)
S22	EMB.EXACT.EXACT("randomization")
S23	EMB.EXACT.EXACT("intermethod comparison")
S24	TI(placebo) OR AB(placebo)
S25	TI(compare OR compared OR comparison) OR AB(compare OR compared OR comparison)
S26	AB(evaluated OR evaluate OR evaluating OR assessed OR assess) AND AB(compare OR compared OR comparing OR comparison)
S27	TI(open NEAR/1 label) OR AB(open NEAR/1 label)
S28	(TI(double OR single OR doubly OR singly) NEAR/1 TI(blind OR blinded OR blindly)) OR (AB(double OR single OR doubly OR singly) NEAR/1 OR AB(blind OR blinded OR blindly))
S29	EMB.EXACT.EXACT("double blind procedure")
S30	TI(parallel NEAR/1 group*) OR AB(parallel NEAR/1 group*)
S31	TI(crossover OR "cross over") OR AB(crossover OR "cross over")
S32	(TI(assign* OR match OR matched OR allocation) NEAR/6 TI(alternate OR group OR groups OR intervention OR interventions OR patient OR patients OR subject OR subjects OR participant OR participants)) OR (AB(assign* OR match OR matched OR allocation) NEAR/6 AB(alternate OR group OR groups OR intervention OR interventions OR patient OR patients OR subject OR subjects OR participant OR participants))
S33	TI(assigned OR allocated) OR AB(assigned OR allocated)
S34	(TI(controlled) NEAR/8 TI(study OR design OR trial)) OR (AB(controlled) NEAR/8 AB(study OR design OR trial))
S35	TI(volunteer OR volunteers) OR AB(volunteer OR volunteers)
S36	EMB.EXACT.EXACT("human experiment")
S37	TI(trial)
S38	S19 OR S20 OR S21 OR S22 OR S23 OR S24 OR S25 OR S26 OR S27 OR S28 OR S29 OR S30 OR S31 OR S32 OR S33 OR S34 OR S35 OR S36 OR S37
S39	(TI(random* NEAR/1 sampl* NEAR/8 ("cross section*" OR questionnaire* OR survey OR surveys OR database OR databases)) OR AB(random* NEAR/1 sampl* NEAR/8 ("cross section*" OR questionnaire* OR survey OR surveys OR database OR databases))) NOT (EMB.EXACT.EXPLODE("comparative study") OR EMB.EXACT.EXPLODE("controlled study") OR TI("randomised controlled") OR AB("randomised controlled") OR TI("randomized controlled") OR AB("randomized controlled") OR TI("randomly assigned") OR AB("randomly assigned"))
S40	EMB.EXACT.EXACT("cross-sectional study") NOT (EMB.EXACT.EXPLODE("randomized controlled trial") OR EMB.EXACT.EXACT("controlled clinical study") OR EMB.EXACT.EXACT("controlled study") OR TI("randomised controlled") OR AB("randomised controlled") OR TI("randomized controlled") OR AB("randomized controlled") OR TI("control group") OR AB("control group") OR TI("control groups") OR AB("control groups"))
S41	(TI("case control*") OR AB("case control*")) AND (TI(random*) OR AB(random*)) NOT (TI("randomised controlled") OR AB("randomised controlled") OR TI("randomized controlled") OR AB("randomized controlled"))
S42	TI("systematic review") NOT TI(trial OR study)
S43	(TI(nonrandom*) OR AB(nonrandom*)) NOT (TI(random*) OR AB(random*))
S44	TI("random field*") OR AB("random field*")
S45	TI("random cluster" NEAR/4 sampl*) OR AB("random cluster" NEAR/4 sampl*)
S46	(AB(review) AND TI(review)) NOT TI(trial)
S47	AB("we searched") AND (TI(review) OR RTYPE(review))
S48	AB("update review")
S49	AB(databases NEAR/5 searched)
S50	((TI(rat OR rats OR mouse OR mice OR swine OR porcine OR murine OR sheep OR lambs OR pigs OR piglets OR rabbit OR rabbits OR cat OR cats OR dog OR dogs OR cattle OR bovine OR monkey OR monkeys OR trout OR marmoset*) OR AB(rat OR rats OR mouse OR mice OR swine OR porcine OR murine OR sheep OR lambs OR pigs OR piglets OR rabbit OR rabbits OR cat OR cats OR dog OR dogs OR cattle OR bovine OR monkey OR monkeys OR trout OR marmoset*)) AND EMB.EXACT.EXACT("animal experiment"))
S51	EMB.EXACT.EXACT("animal experiment") NOT (EMB.EXACT.EXACT("human experiment") OR EMB.EXACT.EXACT("human"))
S52	S39 OR S40 OR S41 OR S42 OR S43 OR S44 OR S45 OR S46 OR S47 OR S48 OR S49 OR S50 OR S51
S53	S38 NOT S52
S54	S5 AND S18 AND S53

**Table 8:** Search strategies: Igaku-Chuo-Zasshi (ICHUSHI)

Searches	Keywords
#1	((危篤/TH or 危篤/TA)) and (PT=会議録除く)
#2	((クリティカルケア/TH or クリティカルケア/TA)) and (PT=会議録除く)
#3	#1 OR #2
#4	((理学療法/TH or 理学療法/TA)) and (PT=会議録除く)
#5	((作業療法/TH or 作業療法/TA)) and (PT=会議録除く)
#6	((理学療法とリハビリテーション医学/TH or 理学療法とリハビリテーション医学/TA)) and (PT=会議録除く)
#7	((運動療法/TH or 運動療法/TA)) and (PT=会議録除く)
#8	((リハビリテーション/TH or リハビリテーション/TA)) and (PT=会議録除く)
#9	((患者ケア計画/TH or 患者ケア計画/TA)) and (PT=会議録除く)
#10	((機能訓練/TA or 運動指導/TA or 運動処方/TA or 運動負荷/TA or 機能療法/TA)) and (PT=会議録除く)
#11	((リハビリ/TA or 社会復帰/TA)) and (PT=会議録除く)
#12	((ケアプラン/TA or 患者医療計画/TA or 療養計画/TA)) and (PT=会議録除く)
#13	#4 OR #5 OR #6 OR #7 OR #8 OR #9 OR #10 OR #11 OR #12
#14	ランダム化比較試験/TH or 準ランダム化比較試験/TH or ランダム化/AL or 無作為化/AL or 比較試験/AL or 臨床試験/AL or プラセボ/AL or 対照/AL or コントロール/AL or 臨床研究/AL
#15	#3 and #13 and #14

**Table 9:** Search strategies: PEDro search strategy

Searches	Keywords
S1	Abstract & Title: "critical care"
S2	Abstract & Title: " critical illness"
S3	Abstract & Title: "critical care"
Method: clinical trial	

**Table 10:** Search strategies: CINAHL search strategy CINAHL: Cumulated Index to Nursing and Allied Health Literature

Searches	Keywords
S1	((MH "Critical Illness+") OR (MH "Critical Care+") OR (TI "critically ill" OR AB "critically ill") OR (TI "Critical Care" OR AB "Critical Care") OR (TI "Critical Illness" OR AB "Critical Illness"))
S2	((MH "physical therapy modalities+") OR (MH "occupational therapy+") OR (MH "Physical and Rehabilitation Medicine+") OR (MH "Exercise Therapy+") OR (MH Rehabilitation+) OR (MH "Patient Care Planning+") OR (TI physiotherap* OR AB physiotherap*) OR (TI "occupational therapy" OR AB "occupational therapy") OR (TI "physical therapy" OR AB "physical therapy") OR (TI "Exercise Therapy" OR AB "Exercise Therapy") OR (TI Rehabilitation OR AB Rehabilitation) OR (TI "Patient Care Planning" OR AB "Patient Care Planning") )
S3	(((MH randomized controlled trials) OR (MH double-blind studies) OR (MH single-blind studies) OR (MH random assignment) OR (MH pretest-posttest design) OR (MH cluster sample) OR (TI (randomised OR randomized)) OR( AB (random*)) OR (TI (trial)) OR (MH (sample size) AND AB (assigned OR allocated OR control)) OR (MH (placebos)) OR( PT (randomized controlled trial)) OR (AB (control W5 group)) OR (MH (crossover design) OR MH (comparative studies))))NOT ((((MH animals+) OR( MH (animal studies)) OR (TI (animal model*))) NOT (MH (human))))
S4	S1 AND S2 AND S3

**Table 11:** Search strategies: The World Health Organization International Clinical Trials Platform Search Portal

Advanced search
Condition: 'Critical Illness' OR 'Critical Care' OR 'Critically Ill' AND Intervention: 'physical therapy modalities' OR 'occupational therapy' OR 'physical and rehabilitation medicine' OR 'exercise therapy' OR rehabilitation OR 'patient care planning' (without synonyms box unchecked)
Recruitment status: 'all'

**Table 12:** Search strategies: ClinicalTrials.gov

Keywords
Condition or disease: 'Critical Illness' OR 'critical care' OR 'critically ill'
Age group: adult (18-64 years), older adult (≥65 years)
Intervention/treatment: 'physical therapy modalities' OR 'occupational therapy' OR 'physical and rehabilitation medicine' OR 'exercise therapy' OR 'rehabilitation' OR 'patient care planning'

A summary of the excluded studies is presented in Table 13.

**Table 13:** Studies excluded in full-text screening ICU: intensive care unit, RCT: randomized controlled trial

First investigator	Journal	Title	Reason for exclusion
Smith DS	Br Med J	Remedial therapy after stroke: a randomised controlled trial	Including patients who were not admitted to the ICU
Dollfus P	J Emerg Med	Rehabilitation following injury to the spinal cord	Not an RCT
Broslawski GE	J Am Osteopath Assoc	Functional abilities of elderly survivors of intensive care	Not an RCT
Niskanen M	Schweiz Med Wochenschr	Quality of life after intensive care	Not an RCT
Hoffmann B	Acta Neurochir Suppl	Incidence and management of complications during posttraumatic early rehabilitation	Not an RCT
Jones C	Crit Care Med	Rehabilitation after critical illness: a randomized, controlled trial	Interventions initiated during hospitalization
Eddleston J	https://trialsearch.who.int/Trial2.aspx?TrialID=ISRCTN38674852	Rehabilitation following critical illness	Interventions initiated during hospitalization
Cuthbertson BH	BMC health services research	A pragmatic randomised, controlled trial of intensive care follow up programmes in improving longer-term outcomes from critical illness. The PRACTICAL study	Interventions for post-ICU follow-up program
Cuthbertson BH	BMJ (Clinical research ed.)	The PRaCTICaL study of nurse led, intensive care follow-up programmes for improving long term outcomes from critical illness: a pragmatic randomised controlled trial	Interventions for post-ICU follow-up program
Garuti G	Rassegna di patologia dell'apparato respiratorio	Home rehabilitation and therapy	Wrong language
Griffiths RD	https://clinicaltrials.gov/show/NCT01063738	Rehabilitating Muscle After Intensive Care	Interventions initiated during hospitalization
Mackintosh S	https://trialsearch.who.int/Trial2.aspx?TrialID=ACTRN12612000972820	Therapist-devised physical rehabilitation programs conducted by the family member(s) of inpatients following an acquired brain injury: a pilot randomised control trial	Including patients who were not admitted to the ICU
(Not applicable)	https://clinicaltrials.gov/show/NCT01770821	Rehabilitation After Intensive Care	Interventions initiated during hospitalization
Walker W	J Intensive Care Soc	Project Post Intensive Care eXercise (PIX): A qualitative exploration of intensive care unit survivors' perceptions of quality of life post-discharge and experience of exercise rehabilitation	Not an RCT
Jensen JF	Intensive Care Med	A recovery program to improve quality of life, sense of coherence and psychological health in ICU survivors: a multicenter randomized controlled trial, the RAPIT study	Interventions for post-ICU follow-up program
Ke H	Chinese nursing research	Effect observation on four stage early activity and rehabilitation exercise therapy for prevention of patients with ICU acquired weakness	Wrong language
Zhao J	Neuropsychological rehabilitation	The effects of cognitive intervention on cognitive impairments after intensive care unit admission	Interventions initiated during hospitalization
Bohart S	Intensive Crit Care Nurs	Recovery programme for ICU survivors has no effect on relatives' quality of life: Secondary analysis of the RAPIT-study	Relatives of intensive care patients as participants
Gilmartin M	Physiotherapy practice & research	Intensive care discharge facilitation using the REhabilitation after Critical illness Assisted discharge Pack (RECAP) model: a pilot randomized controlled trial	Interventions initiated during hospitalization
Khan S	Trials	Mobile critical care recovery program (m-CCRP) for acute respiratory failure survivors: study protocol for a randomized controlled trial	Intervention for individualized care plan
Wang S	Trials	Improving Recovery and Outcomes Every Day after the ICU (IMPROVE): study protocol for a randomized controlled trial	Protocol without results
Berry MJ	J Crit Care	The relationship between self-report and performance-based measures of physical function following an ICU stay	Interventions initiated during hospitalization
Ferguson K	J Intensive Care Med	Patients' Perceptions of an Exercise Program Delivered Following Discharge From Hospital After Critical Illness (the Revive Trial)	Not an RCT
Liang Z	https://clinicaltrials.gov/show/NCT03885687	Exercise With Music for ICU Survivors	Intervention for music
Boehm LM	https://clinicaltrials.gov/show/NCT03926533	Telehealth-Enhanced Patient-Oriented Recovery Trajectory After ICU	Intervention for telehealth ICU recovery program
Yu HL	Neurol Res	Effect of a novel designed intensive patient care program on cognitive impairment, anxiety, depression as well as relapse free survival in acute ischemic stroke patients: a randomized controlled study	Including patients who were not admitted to the ICU
Liang Z	American journal of respiratory and critical care medicine	Feasibility and acceptability of a self-managed exercise to rhythmic music intervention for ICU survivors	Congress abstract
Jiang M	Ann Palliat Med	Effect analysis of kinetic energy progressive exercise in patients with acute myocardial infarction after percutaneous coronary intervention: a randomized trial	Interventions initiated during hospitalization
Major M	Crit Care	Feasibility of a home-based interdisciplinary rehabilitation program for patients with Post-Intensive Care Syndrome: the REACH study	Not an RCT
Rousseau AF	https://clinicaltrials.gov/show/NCT05111275	Cognitive Exercises in Survivors of a Prolonged ICU Stay	Ongoing study
O'Neill B	https://trialsearch.who.int/Trial2.aspx?TrialID=ISRCTN11266403	Remote rehabilitation after intensive care	Ongoing study
Liang Z	Biol Res Nurs	Self-Managed Music-Guided Exercise Intervention Improved Upper and Lower Extremity Muscle Strength for ICU Survivors-A Pilot Randomized Controlled Study	Interventions initiated during hospitalization
Boerma EC	https://clinicaltrials.gov/show/NCT05182086	Improving Recovery After Critical Illness	Ongoing study
Wischmeyer P	https://clinicaltrials.gov/show/NCT05218083	REmotely Monitored, Mobile Health Supported High Intensity Interval Training to Improve Functional Recovery of Survivors of COVID-19 Critical Illness	Ongoing study
Platz T	BMC Neurol	Optimizing home-based long-term intensive care for neurological patients with neurorehabilitation outreach teams - protocol of a multicenter, parallel-group randomized controlled trial (OptiNIV-Study)	Protocol without results
Parker AM	https://clinicaltrials.gov/show/NCT03431493	A Pilot, Feasibility Randomized Controlled Trial of a Behavioral Activation And Rehabilitation Intervention To Improve Psychological And Physical Impairments In Acute Respiratory Failure Survivors	Ongoing study
Tate J	https://clinicaltrials.gov/show/NCT03972384	A Problem Solving Intervention for Post-ICU Cognitive Impairment in Older Adults	Ongoing study
Wischmeyer P	https://clinicaltrials.gov/show/NCT04664101	REmotely Monitored, Mobile Health-Supported High Intensity Interval Training After COVID-19 Critical Illness (REMM-HIIT-COVID-19)	Ongoing study
Fernandez RS	Aust Crit Care	A pilot randomised controlled trial comparing a health-related lifestyle self- management intervention with standard cardiac rehabilitation following an acute cardiac event: Implications for a larger clinical trial	Including patients who were not admitted to the ICU
Connolly B	Difficulties of patient recruitment to a post critical illness rehabilitation programme	American Journal of Respiratory and Critical Care Medicine	Congress abstract
Goodman J	Critical Care	Project PIX (Post Intensive care eXercise): Impact on physical fitness and focus group analysis of quality of life following exercise rehabilitation	Congress abstract
Battle C	Journal of the Intensive Care Society	Early results of a six-week supervised exercise programme in post-ICU patients	Congress abstract
Jones C	J Crit Care	Improving rehabilitation after critical illness through outpatient physiotherapy classes and essential amino acid supplement: A randomized controlled trial	Interventions initiated during hospitalization
Verceles AC	American Journal of Respiratory and Critical Care Medicine	Improved weaning success and discharge home with a multimodal rehabilitation program in older patients with post ICU syndrome	Congress abstract
Patsaki I	J Crit Care	Effect of neuromuscular stimulation and individualized rehabilitation on muscle strength in Intensive Care Unit survivors: A randomized trial.	Interventions initiated during hospitalization
Battle C	Physiotherapy	Supervised exercise rehabilitation in survivors of critical illness: a randomised controlled trial	Congress abstract
Orwelius L	Intensive Care Medicine Experimental	Evaluate efficacy of tele-yoga on multiple outcomes after a period of critical illness	Congress abstract
Xue F	Indian Journal of Pharmaceutical Sciences	Rehabilitation therapy for better control of critically ill patients	Interventions initiated during hospitalization
Liguori S	Front Neurol	Rehabilitation of Neuromuscular Diseases During COVID-19: Pitfalls and Opportunities	Not an RCT
McGregor G	Trials	Rehabilitation Exercise and psycholoGical support After covid-19 InfectioN' (REGAIN): a structured summary of a study protocol for a randomised controlled trial	Including patients who were not admitted to the ICU
Castelli L	J Clin Med	The Role of Technological Rehabilitation in Patients with Intensive Care Unit Weakness: A Randomized Controlled Pilot Study	Interventions for non-immersive virtual reality combined with focal muscle vibration
Dong Q	Aust Crit Care	Effects of early cognitive rehabilitation training on cognitive function and quality of life in critically ill patients with cognitive impairment: A randomised controlled trial	Interventions initiated during hospitalization
Orvelius L	Intensive care medicine experimental	Effects of tele-yoga on physical and psychological outcomes in patients with long-term critical illness-a randomised controlled trial	Congress abstract
Li PWC	https://clinicaltrials.gov/ct2/show/NCT06117761	Combined Activity and Cognitive Intervention to Optimize Recovery From Critical Illness in ICU Survivors: COMBAT-ICU Trial	Ongoing study
(Not applicable)	https://clinicaltrials.gov/ct2/show/NCT06159868	Physiotherapy and Optimised Enteral Nutrition In the Post-acute Phase of Critical Illness (PHOENIX): A Randomised Controlled Feasibility Trial	Interventions initiated during hospitalization

Risk of bias summary and graphs are shown in Figure 10. 
